# Supplementary material for: A Likelihood Approach for Real-Time Calibration of Stochastic Compartmental Epidemic Models
Source: PLoS Comput Biol. 2017 Jan 17;13(1):e1005257. doi: 10.1371/journal.pcbi.1005257 (PMC5240920; doi:10.1371/journal.pcbi.1005257)
Supplement: S1 File — (TAR.GZ) [file pcbi.1005257.s014.tar.gz › HSPH_Online-SI-Revision/MSS10/n1-extreme/n1-extreme_NEW_table-8.pdf]

1	R0	{2.95019, 14.2854, 0.1}	{1.90936, 13.8612, 0.48}	{{1.14344, 8.71343, 0.}, {2.7499, 19.1478, 1.}}	{35.3437, 24.6775, 502.}	inf	3.	3.36398	{1.37328, 6.2897}	51.4644	0.20694	newly	0.1	3.57	{0., 6.}	3498.	1.19616	Reff	2.93839	1.90049	{1.14083, 2.74254}	35.3784	ar	924.	576.72	{3., 921.}	37.8831
2	R0	{2.67565, 16.946, 0.1}	{2.34191, 14.7563, 0.1}	{{1.81768, 8.25876, 0.}, {2.93978, 19.9254, 0.}}	{16.3215, 21.5024, 182.}	inf	30.	21.1589	{7.98211, 37.5607}	40.971	0.262721	newly	15.	14.24	{4., 25.}	44.	0.229372	Reff	2.55525	2.25428	{1.76285, 2.80839}	15.6251	ar	926.	843.56	{738., 935.}	9.39525
3	R0	{2.40066, 16.6543, 0.1}	{2.1588, 13.3126, 1.03}	{{1.5102, 6.00596, 0.}, {2.86001, 18.8445, 2.}}	{18.4671, 25.3992, 992.}	inf	14.	11.7712	{3.85261, 21.263}	41.6723	0.239749	newly	8.	9.63	{3., 17.}	62.875	0.263396	Reff	2.34544	2.11204	{1.4908, 2.75311}	18.1218	ar	916.	768.	{540., 915.}	16.5808
4	R0	{1.9294, 15.4361, 0.1}	{1.34345, 11.1444, 1.5}	{{1.0306, 4.00571, 0.}, {1.84468, 18.765, 3.}}	{31.0553, 37.8981, 1462.}	inf	10.	4.36079	{1.33354, 9.9762}	59.821	0.47714	newly	5.	4.49	{0., 11.}	77.8	0.51363	Reff	1.89467	1.32655	{1.01858, 1.8248}	30.7079	ar	796.	259.12	{10., 755.}	67.8342
5	R0	{2.92859, 19.5393, 0.1}	{1.28062, 17.3318, 0.32}	{{1.13754, 16.005, 0.}, {1.36512, 19.4013, 1.}}	{56.2719, 11.2995, 366.}	inf	28.	7.94709	{3.26688, 11.6714}	71.6175	0.606363	newly	16.	3.39	{1., 6.}	79.5625	0.856007	Reff	2.7968	1.24853	{1.10909, 1.33326}	55.3585	ar	927.	316.9	{42., 503.}	65.8145
6	R0	{1.79622, 12.3148, 0.1}	{2.21818, 14.1865, 0.52}	{{1.62191, 8.4085, 0.}, {2.76795, 19.0355, 1.}}	{27.6209, 29.4143, 538.}	inf	35.	42.3107	{17.694, 66.0979}	45.0998	0.18934	newly	31.	27.29	{14., 43.}	30.9355	0.161434	Reff	1.6651	2.03401	{1.54399, 2.45383}	25.7892	ar	734.	819.28	{679., 921.}	16.5259
7	R0	{2.45855, 17.1776, 1.}	{2.13556, 12.0688, 0.43}	{{1.38815, 6.48739, 0.}, {2.91001, 18.788, 1.}}	{21.4011, 32.7703, 79.}	inf	24.	6.08101	{1.5747, 13.1302}	74.6625	0.721521	newly	13.	6.01	{1., 16.}	70.6923	0.665333	Reff	2.38971	2.11244	{1.38094, 2.86139}	21.0083	ar	871.	702.55	{10., 935.}	23.225
8	R0	{2.20009, 15.519, 2.}	{1.83247, 13.4232, 1.43}	{{1.38815, 6.71734, 0.}, {2.26107, 18.9032, 2.}}	{19.4909, 24.2696, 38.5}	inf	26.	48.2645	{22.6065, 78.7891}	93.1612	0.260747	newly	18.	28.53	{15., 43.}	66.2778	0.21023	Reff	2.00208	1.62055	{1.27952, 1.96534}	20.2457	ar	839.	697.73	{475., 870.}	18.7139
9	R0	{2.06493, 9.6414, 1.}	{1.98468, 4.57731, 0.11}	{{1.22544, 2.19254, 0.}, {2.91946, 6.48739, 0.}}	{28.135, 55.081, 97.}	inf	4.	10.7485	{3.15628, 20.3276}	178.935	0.386093	newly	3.	47.88	{6., 129.}	1500.	0.977223	Reff	2.04222	1.94699	{1.21082, 2.85439}	27.6685	ar	777.	640.42	{36., 935.}	31.1454
10	R0	{2.60775, 19.6936, 1.}	{1.7289, 14.6697, 0.25}	{{1.40261, 9.98152, 0.}, {2.2194, 19.1478, 1.}}	{33.7013, 25.5105, 81.}	inf	29.	16.8732	{6.08739, 29.8991}	47.5292	0.342003	newly	16.	9.12	{3., 16.}	47.375	0.342653	Reff	2.47997	1.66322	{1.35641, 2.0854}	32.9338	ar	889.	652.97	{466., 811.}	26.5568
11	R0	{1.99168, 8.13555, 1.}	{1.77729, 10.7852, 0.93}	{{1.10202, 4.02511, 0.}, {2.52368, 18.2812, 2.}}	{23.42, 58.9084, 75.}	inf	4.	6.4404	{1.91674, 12.5158}	93.1381	0.285274	newly	3.	7.65	{1., 16.}	191.	0.44783	Reff	1.97375	1.75583	{1.09046, 2.49466}	23.2672	ar	772.	553.67	{10., 889.}	36.693
12	R0	{1.79304, 9.18941, 0.1}	{1.92223, 13.8493, 0.51}	{{1.20997, 6.71734, 0.}, {2.88523, 19.3966, 2.}}	{28.6499, 58.7897, 532.}	inf	3.	2.72067	{1.20127, 4.61448}	39.3138	0.190855	newly	4.	2.35	{0., 5.}	68.25	0.64688	Reff	1.78587	1.91474	{1.20722, 2.87434}	28.6017	ar	725.	498.83	{3., 929.}	49.3007
13	R0	{1.84016, 18.3431, 1.}	{1.88588, 15.3161, 0.79}	{{1.55644, 10.5556, 0.}, {2.22832, 18.8228, 1.}}	{9.89406, 17.4549, 41.}	inf	36.	28.0729	{13.8709, 41.7524}	32.8605	0.193667	newly	12.	15.65	{8., 24.}	47.0833	0.168578	Reff	1.71319	1.76889	{1.45474, 2.07509}	9.69978	ar	762.	736.85	{596., 843.}	9.76772
14	R0	{1.94221, 9.85971, 1.}	{2.35827, 12.6315, 0.62}	{{1.87927, 9.06807, 0.}, {2.93488, 18.0404, 1.}}	{25.5638, 36.2527, 46.}	inf	16.	29.9863	{16.0023, 44.2478}	92.1866	0.264832	newly	13.	24.95	{11., 38.}	99.3077	0.278029	Reff	1.86646	2.23123	{1.78507, 2.75742}	23.8856	ar	805.	844.82	{770., 934.}	11.9354
15	R0	{1.78578, 12.0541, 0.1}	{1.96904, 16.0354, 0.07}	{{1.70497, 13.7237, 0.}, {2.27582, 18.9086, 0.}}	{13.9019, 35.9773, 158.}	inf	16.	37.9201	{20.9749, 57.8037}	140.47	0.358284	newly	14.	19.19	{10., 27.}	51.0714	0.185249	Reff	1.68578	1.81308	{1.57531, 2.06747}	11.9471	ar	732.	767.65	{671., 869.}	11.6243
16	R0	{1.94463, 15.6503, 2.}	{1.86474, 14.8677, 0.32}	{{1.15921, 9.75208, 0.}, {2.6365, 19.4475, 1.}}	{24.1837, 19.9313, 84.}	inf	8.	4.1774	{1.16896, 7.32672}	58.4577	0.422985	newly	4.	2.96	{0., 7.}	76.5	0.582263	Reff	1.92518	1.85239	{1.15571, 2.62448}	24.102	ar	757.	556.64	{4., 903.}	38.3065
17	R0	{2.08901, 16.4314, 0.1}	{1.80823, 15.4503, 0.27}	{{1.24145, 10.3394, 0.}, {2.27582, 19.3966, 1.}}	{18.8495, 16.7264, 324.}	inf	17.	7.87126	{1.96347, 14.1648}	56.9245	0.448331	newly	8.	4.45	{1., 9.}	54.875	0.463824	Reff	2.03887	1.77989	{1.23033, 2.24246}	18.3116	ar	826.	599.74	{11., 863.}	29.4189
18	R0	{2.76419, 18.56, 2.}	{1.92709, 9.45514, 0.97}	{{1.22544, 3.26445, 0.}, {2.75598, 16.4899, 2.}}	{31.3827, 49.5801, 57.5}	inf	14.	10.6048	{2.92526, 18.2329}	46.0009	0.299564	newly	7.	14.43	{2., 31.}	132.714	0.362431	Reff	2.70338	1.88902	{1.20927, 2.68367}	31.17	ar	920.	624.79	{75., 915.}	32.538
19	R0	{2.3821, 17.1399, 2.}	{2.14977, 10.2333, 0.61}	{{1.38815, 4.06661, 0.}, {2.91001, 16.8599, 2.}}	{21.2179, 41.3309, 73.5}	inf	12.	5.2435	{1.73157, 10.1226}	57.7777	0.448927	newly	10.	9.44	{1., 19.}	84.	0.499827	Reff	2.34637	2.13137	{1.37787, 2.87435}	21.0528	ar	883.	680.51	{6., 936.}	26.1438
20	R0	{2.78314, 19.6127, 0.1}	{1.92938, 13.3369, 0.44}	{{1.44762, 5.58477, 0.}, {2.2277, 17.7758, 1.}}	{30.676, 31.9986, 454.}	inf	70.	44.9252	{20.8747, 66.3498}	38.0298	0.249045	newly	32.	29.96	{18., 42.}	27.5	0.133952	Reff	2.44916	1.74196	{1.3541, 1.99037}	28.8755	ar	943.	742.98	{521., 862.}	21.211
21	R0	{1.85124, 14.6794, 0.1}	{2.26783, 15.1602, 0.53}	{{1.71608, 10.1363, 0.}, {2.81891, 18.9032, 1.}}	{26.47, 20.6542, 540.}	inf	18.	23.595	{9.38001, 35.6705}	51.633	0.198352	newly	15.	16.22	{5., 25.}	47.3333	0.204951	Reff	1.78274	2.16917	{1.67416, 2.67728}	25.5264	ar	730.	829.19	{720., 917.}	19.0288
22	R0	{2.1028, 14.9852, 2.}	{2.03419, 11.8697, 0.61}	{{1.38815, 6.0871, 0.}, {2.73108, 17.7758, 2.}}	{20.1934, 28.2545, 69.5}	inf	26.	16.8051	{6.16077, 30.3345}	43.5272	0.301172	newly	17.	13.46	{4., 22.}	39.5294	0.244396	Reff	2.01659	1.96614	{1.35644, 2.6282}	19.6832	ar	840.	724.7	{427., 917.}	18.65
23	R0	{1.86601, 10.2157, 1.}	{1.78589, 13.5022, 0.7}	{{1.12882, 8.31782, 0.}, {2.52588, 18.727, 2.}}	{23.7733, 41.054, 74.}	inf	8.	4.16178	{1.36802, 8.13028}	55.8282	0.408116	newly	6.	3.12	{0., 8.}	65.3333	0.618656	Reff	1.84548	1.77239	{1.12417, 2.4915}	23.6947	ar	747.	552.06	{6., 886.}	36.9665
24	R0	{2.34032, 10.0992, 0.1}	{1.75872, 13.4879, 0.38}	{{1.11927, 6.71734, 0.}, {2.64721, 19.0355, 1.}}	{30.3773, 45.8287, 422.}	inf	2.	3.05412	{1.30653, 5.47366}	71.7864	0.202035	newly	1.	2.57	{0., 7.}	197.	0.500555	Reff	2.3333	1.75093	{1.11661, 2.63277}	30.35	ar	887.	454.78	{3., 916.}	49.9842
25	R0	{1.91548, 15.3401, 1.}	{1.74382, 13.4012, 0.6}	{{1.16434, 7.26549, 0.}, {2.2277, 18.7084, 2.}}	{18.0716, 23.2846, 70.}	inf	10.	6.0505	{1.88473, 10.8521}	47.6071	0.329931	newly	8.	4.16	{1., 8.}	57.75	0.478602	Reff	1.88292	1.72029	{1.15485, 2.1948}	17.8317	ar	765.	582.64	{16., 846.}	30.366
26	R0	{2.75283, 10.6721, 0.1}	{1.95488, 12.9868, 0.51}	{{1.13754, 6.28417, 0.}, {2.73108, 18.2812, 2.}}	{30.0379, 35.055, 534.}	inf	2.	5.14518	{1.55436, 9.28587}	166.207	0.357568	newly	1.	4.02	{0., 8.}	322.	0.567705	Reff	2.73907	1.93794	{1.13282, 2.70286}	30.141	ar	926.	642.47	{12., 918.}	30.8801
27	R0	{2.08367, 19.7849, 1.}	{1.5719, 12.0913, 1.97}	{{1.24271, 4.02511, 0.}, {1.87294, 19.0269, 3.}}	{24.9014, 38.8863, 135.}	inf	51.	21.3193	{6.89075, 33.6201}	58.8858	0.465757	newly	27.	15.17	{6., 27.}	50.7778	0.352145	Reff	1.89822	1.47665	{1.17638, 1.74186}	22.5151	ar	833.	572.12	{142., 766.}	31.383
28	R0	{2.64334, 17.1765, 0.1}	{2.43021, 10.1924, 0.39}	{{1.93419, 6.00596, 0.}, {2.93371, 12.8326, 1.}}	{13.1075, 40.8678, 422.}	inf	29.	25.3989	{6.99639, 42.9916}	41.8587	0.249079	newly	16.	29.25	{8., 48.}	103.563	0.300409	Reff	2.5191	2.32154	{1.84511, 2.71018}	12.4405	ar	934.	849.75	{749., 936.}	9.22805
29	R0	{2.87008, 13.3872, 0.1}	{1.86411, 15.7202, 0.29}	{{1.45954, 10.9674, 0.}, {2.27582, 19.2972, 1.}}	{35.0501, 24.6849, 340.}	inf	6.	11.0315	{4.26069, 17.7755}	95.9854	0.281796	newly	4.	5.87	{2., 10.}	69.75	0.266041	Reff	2.82129	1.82233	{1.43602, 2.22115}	35.4079	ar	941.	682.93	{344., 862.}	27.4251
30	R0	{2.06105, 19.6275, 1.}	{1.68044, 11.3998, 0.57}	{{1.52414, 9.52864, 0.}, {1.78225, 13.8621, 2.}}	{18.6035, 41.9194, 81.}	inf	103.	71.5642	{49.627, 87.8459}	33.1326	0.181637	newly	49.	48.81	{33., 65.}	19.6939	0.0878611	Reff	1.55609	1.31983	{1.23663, 1.40971}	15.2532	ar	747.	670.27	{581., 750.}	11.6774
31	R0	{2.30764, 13.435, 2.}	{2.01284, 13.9218, 0.45}	{{1.13754, 8.33653, 0.}, {2.7499, 19.5128, 2.}}	{21.8297, 27.0381, 79.5}	inf	14.	4.69108	{1.6628, 8.25874}	66.7284	0.552015	newly	11.	4.02	{1., 8.}	74.1818	0.746771	Reff	2.26841	1.99673	{1.13093, 2.73253}	21.6206	ar	877.	604.59	{6., 929.}	33.4584
32	R0	{2.30565, 11.9237, 0.1}	{2.35313, 10.0049, 0.56}	{{1.47608, 4.56746, 0.}, {2.96398, 14.464, 2.}}	{18.6398, 28.8586, 578.}	inf	8.	13.1606	{4.44197, 23.3163}	86.5659	0.273562	newly	11.	16.54	{4., 32.}	80.9091	0.273632	Reff	2.26876	2.30108	{1.45653, 2.87517}	18.1095	ar	853.	815.54	{570., 947.}	13.1536
33	R0	{2.24031, 18.582, 1.}	{1.18302, 16.868, 0.3}	{{1.02099, 13.1236, 0.}, {1.36512, 19.4013, 1.}}	{47.1939, 11.4158, 72.}	inf	23.	3.23836	{1.40516, 5.74859}	85.9202	0.915684	newly	10.	1.63	{0., 3.}	83.7	1.06745	Reff	2.17982	1.17426	{1.01527, 1.35414}	46.1302	ar	864.	170.9	{6., 502.}	80.2199
34	R0	{2.33306, 15.9759, 0.1}	{2.07077, 11.1263, 0.84}	{{1.28596, 3.83841, 0.}, {2.86501, 18.1089, 2.}}	{22.7391, 36.1201, 818.}	inf	16.	11.4802	{3.05285, 21.4549}	45.8769	0.301222	newly	12.	12.97	{2., 26.}	66.5833	0.327097	Reff	2.27473	2.02564	{1.26228, 2.79844}	22.3061	ar	854.	716.45	{271., 926.}	21.1628
35	R0	{2.48357, 18.3573, 1.}	{1.65556, 15.3945, 0.33}	{{1.1315, 9.75208, 0.}, {2.23915, 19.5412, 1.}}	{34.7339, 19.3515, 73.}	inf	17.	3.08827	{1.30779, 5.12508}	81.8337	0.815064	newly	9.	2.88	{0., 4.}	92.2222	0.996581	Reff	2.43887	1.64824	{1.12626, 2.23393}	33.9684	ar	835.	442.87	{4., 849.}	48.6647
36	R0	{2.59548, 19.9046, 0.1}	{1.88527, 13.6834, 0.79}	{{1.5102, 9.08517, 0.}, {2.20181, 17.7758, 2.}}	{27.6288, 31.2628, 774.}	inf	18.	15.1717	{6.54582, 24.5614}	33.2955	0.188985	newly	13.	9.74	{3., 17.}	41.8462	0.256119	Reff	2.50983	1.82706	{1.47474, 2.12968}	27.4486	ar	863.	707.59	{478., 860.}	18.6732
37	R0	{2.29641, 16.9868, 0.1}	{1.56904, 13.3227, 0.29}	{{1.27063, 10.7271, 0.}, {1.80508, 18.4377, 1.}}	{31.6741, 24.4997, 336.}	inf	21.	16.5286	{4.48541, 35.3991}	46.2794	0.282942	newly	10.	9.58	{3., 19.}	48.	0.281235	Reff	2.18159	1.49668	{1.23065, 1.69678}	31.3948	ar	877.	553.93	{166., 723.}	36.8381
38	R0	{2.65743, 15.4569, 1.}	{2.52233, 15.9443, 0.12}	{{2.02235, 12.644, 0.}, {2.96797, 18.8857, 1.}}	{12.1945, 15.2056, 90.}	inf	29.	27.5706	{10.3446, 44.2734}	32.6745	0.169829	newly	16.	16.73	{8., 28.}	35.9375	0.167502	Reff	2.52987	2.4021	{1.97131, 2.79508}	11.4156	ar	897.	884.13	{805., 942.}	5.00446
39	R0	{2.67953, 14.5132, 2.}	{1.64242, 15.0105, 0.37}	{{1.08432, 9.63533, 0.}, {2.30907, 19.4013, 1.}}	{39.2062, 24.0599, 81.5}	inf	2.	3.16324	{1.15368, 5.69767}	74.9095	0.226277	newly	1.	2.33	{0., 4.}	167.	0.417465	Reff	2.67149	1.63514	{1.08048, 2.30033}	39.2632	ar	894.	376.61	{3., 866.}	58.3031
40	R0	{1.94594, 11.928, 1.}	{2.42583, 13.0408, 0.51}	{{1.93539, 8.81055, 0.}, {2.93371, 18.0404, 1.}}	{27.051, 24.1715, 57.}	inf	31.	29.1702	{12.0868, 44.8947}	32.808	0.174592	newly	31.	23.35	{10., 38.}	38.8065	0.246483	Reff	1.84475	2.30147	{1.86443, 2.74659}	27.0273	ar	796.	857.69	{748., 937.}	12.3606
41	R0	{1.66297, 10.0047, 0.1}	{1.37904, 9.18856, 1.62}	{{1.08063, 2.5364, 0.}, {1.64587, 19.5316, 4.}}	{17.1033, 65.7345, 1612.}	inf	25.	20.4749	{3.43608, 44.3262}	51.0526	0.333592	newly	18.	22.99	{6., 47.}	70.2778	0.264408	Reff	1.51995	1.26455	{1.00694, 1.47442}	16.8032	ar	716.	434.14	{96., 673.}	39.3799
42	R0	{2.91568, 16.4453, 1.}	{1.87688, 14.1763, 0.41}	{{1.13754, 8.56054, 0.}, {2.70795, 19.3966, 1.}}	{35.7618, 21.9716, 77.}	inf	9.	2.86605	{1.34531, 5.34863}	68.155	0.548025	newly	8.	2.09	{0., 5.}	76.375	0.867017	Reff	2.88652	1.86927	{1.13387, 2.69786}	35.416	ar	919.	527.78	{3., 921.}	42.7922
43	R0	{2.55898, 15.8889, 0.1}	{1.79161, 14.625, 0.28}	{{1.10202, 8.71343, 0.}, {2.49092, 19.1478, 1.}}	{31.2873, 19.9446, 332.}	inf	3.	3.46943	{1.42137, 6.49986}	56.7143	0.216013	newly	3.	3.04	{0., 6.}	84.	0.504334	Reff	2.54619	1.78119	{1.09354, 2.46917}	31.2641	ar	904.	517.38	{4., 898.}	43.1637
44	R0	{2.28649, 15.9017, 0.1}	{2.24767, 15.4325, 0.24}	{{1.71608, 10.6804, 0.}, {2.93978, 19.5406, 1.}}	{17.0017, 18.121, 300.}	inf	13.	11.6753	{3.89141, 20.2713}	41.9788	0.228613	newly	10.	7.39	{2., 13.}	46.9	0.306162	Reff	2.24076	2.20404	{1.70025, 2.85236}	16.7189	ar	880.	801.08	{652., 934.}	12.3159
45	R0	{2.65121, 18.6339, 0.1}	{2.01056, 11.3751, 0.58}	{{1.22544, 5.15387, 0.}, {2.83858, 18.6082, 2.}}	{27.0597, 39.7147, 600.}	inf	4.	4.95686	{1.79406, 8.70921}	64.5878	0.239277	newly	1.	8.31	{1., 15.}	747.	0.671943	Reff	2.6353	1.99565	{1.21369, 2.81929}	27.0055	ar	928.	575.45	{5., 934.}	38.3157
46	R0	{2.52291, 18.0251, 2.}	{1.38111, 12.1564, 1.38}	{{1.05515, 5.15387, 0.}, {1.88607, 18.8857, 3.}}	{45.7015, 34.4963, 51.}	inf	13.	4.47359	{1.60175, 8.34591}	69.0451	0.58138	newly	3.	4.18	{1., 11.}	99.3333	0.411922	Reff	2.4775	1.36699	{1.04717, 1.86556}	45.2245	ar	894.	322.5	{8., 772.}	64.0783
47	R0	{2.22618, 17.1225, 0.1}	{1.94667, 11.232, 0.87}	{{1.13726, 5.30671, 0.}, {2.60786, 18.8445, 2.}}	{22.4587, 37.4777, 854.}	inf	9.	12.8192	{2.78476, 23.4658}	76.5697	0.278064	newly	3.	11.77	{3., 22.}	301.	0.526648	Reff	2.18388	1.89841	{1.12107, 2.53536}	21.9956	ar	850.	647.44	{24., 906.}	27.5035
48	R0	{2.04405, 16.073, 1.}	{2.43814, 11.5156, 1.09}	{{1.98388, 6.16453, 0.}, {2.98125, 17.2484, 2.}}	{22.2751, 32.5196, 79.}	inf	18.	31.0155	{12.7154, 46.2814}	82.463	0.254506	newly	12.	31.92	{13., 57.}	170.167	0.377542	Reff	1.96229	2.30621	{1.87883, 2.75441}	20.6006	ar	779.	866.63	{770., 935.}	13.1617
49	R0	{2.84396, 18.097, 1.}	{2.00974, 12.3929, 0.61}	{{1.13754, 6.48739, 0.}, {2.7499, 18.4042, 2.}}	{29.8047, 32.8494, 75.}	inf	14.	3.81757	{1.33172, 6.52105}	72.7316	0.635777	newly	11.	6.75	{0., 8.}	100.455	0.76992	Reff	2.79846	1.99761	{1.13336, 2.73059}	29.2217	ar	952.	614.14	{5., 927.}	35.4895
50	R0	{1.85341, 18.0975, 4.}	{1.86901, 6.77927, 0.57}	{{1.24751, 2.5364, 0.}, {2.8186, 15.191, 2.}}	{26.8539, 62.7395, 85.75}	inf	61.	34.5366	{8.6133, 64.2483}	47.6492	0.368643	newly	26.	58.68	{23., 95.}	136.308	0.345176	Reff	1.67178	1.72513	{1.17487, 2.52305}	25.3456	ar	754.	648.94	{285., 924.}	26.7401
